# Menkes kinky hair disease: A case report

**DOI:** 10.1186/1757-1626-1-158

**Published:** 2008-09-18

**Authors:** Asok K Datta, Taraknath Ghosh, Kaustav Nayak, Mrinalkanti Ghosh

**Affiliations:** 1 Department of Pediatrics, Burdwan Medical College, Burdwan-713101, West Bengal, India; 2 Department of Radiology Burdwan Medical College, Burdwan-713101, West Bengal, India

## Abstract

An eight month old male infant with protein energy malnutrition was admitted in the hospital with the history of repeated attacks of convulsion since four months of age. He was also suffering from frequent attacks of cough and cold since 6 months of age which was marked prior to admission. The infant had fair complexion, sparse fuzzy wooly hair with marked trunkal hypotonia. He had also mental retardation. Serum copper and ceruloplasmin levels were low, MRI showed prominent extraaxial spaces with gliosis, MR angiography revealed tortuosity of cerebral vessels. Microscopic examination of hair revealed pili torti. The patient was diagnosed as Menkes disease and treated symptomatically. For lack of facilities we were not able to do genetic study.

## Introduction

Menkes disease is an X-linked lethal multi system disorder caused by disturbances of copper distribution in different tissues due to mutation of p ATPase7 gene. The estimated prevalence of the disease is 1 in 100000 to 1 in 250000 [1]. The affected individual suffers from malfunction of copper containing enzymes resulting in multi systemic disturbances. Nervous system problems include gross mental retardation, convulsions, cortical atrophy, asymptomatic subdural effusion, grosss trunkal hypotonia and progressive neurological deterioration, Vascular problems with weak collagen tissues causes easy breakability, connective tissue abnormality gives rise to characteristics steel, fuzzy, wooly, sparse hair with easy pluckability [2].

The bones are osteopenic. There are chances of recurrent infections and as a result the infant fails to thrive and malnutrition is a common finding [3]. The infant usually die within 3–4 years of age. In 1962, Menkes first described the syndrome and Drank et.el noted the association with copper metabolism [4]. The affected gene was cloned in 1993.

## Case report

An eight month old male hindu tribal infant from a rural area Tatarpur, Memary, Burdwan, a product of nonconsanguineous marriage, admitted in the hospital with the chief complaint of recurrent attacks of convulsion since four months of age, recurrent respiratory difficulty since six months of age which was aggravated for last seven days and associated with fever. The mother also informed that her baby was lagging in growth and development in comparison to the other children of same age and sex in the community. The child had admitted twice before with bronchopneumonia. The infant has three brothers. Eldest one is 5 years, middle one died at fifteen days after birth. They live in a nuclear family in a mud built 2 rooms house with no sanitary latrine, drink water from tube-well, take bath in pond, parents are labourer with monthly income approximate rupees one thousand only.

**Figure 1:**
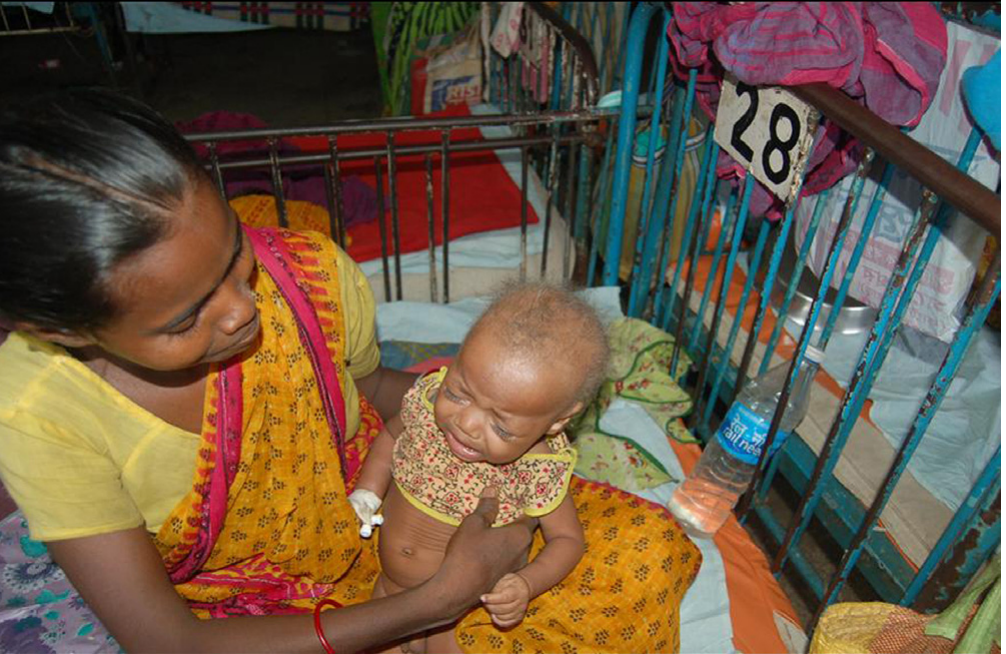
It shows the phenotypic appearance of the baby – the characteristics steel fuzzy sparse hair, fair complexion, The chubby cheeks, irritable baby.

The baby was exclusively breastfed for 4 months, weaning done with suji, biscuits, rice, dal etc which are continuing till now. On examination the baby was conscious but irritable, fair complexion chubby cheeks, light coloured steel sparse wooly hair with easy pluckability (Figure 1). Anthropometric measurement revealed weight 4.5 kg, length 63 cm, head circumference 40.5 cm, chest circumference 36.5 cm, there was mild pallor, skull, spines and bones were normal. pulse 90/min, B.P. 70/50 mm Hg, respiratory rate 55/min, temperature – normal. Central nervous system examination revealed repeated myoclonic seizures of the limbs, Limbs were hypertonic whereas the trunk was hypotonic, generalised muscle wasting present with power grade 3/5 Examination of Respiratory system showed features of pneumonia. Other systems appeared normal.

**Figure 2:**
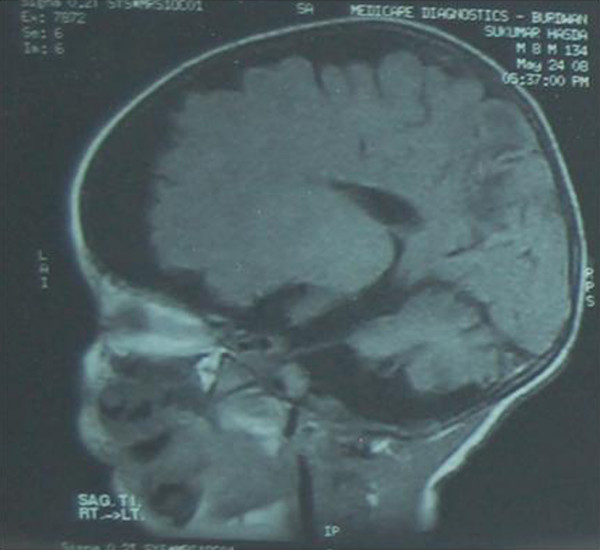
The MRI study of the brain revealed prominent bilateral extra-axial C.S.F. spaces with gliosis in both posterior parieto-occipital area with prominent left lateral ventricle and cerebellar folias.

**Figure 3:**
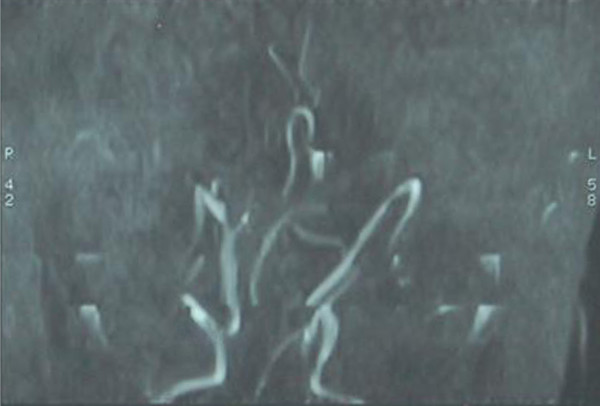
MR angiography reveals tortuosity of the cerebral vessels with hairpin like bending.

**Figure 4:**
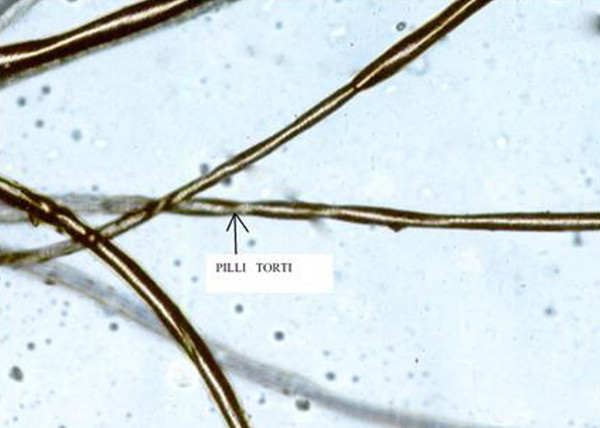
Microscopic examination of hair revealed classical sign of pili torti.

On investigation the infant was found suffering from mild anemia (Hb = 9 gm%), total leucocyte count was increased 16000/dl due to chest infection, cerebrospinal fluid study was normal, chest X-ray showed pneumonitis and the ribs were found osteopenic. The skeletal survey of the limb bones also showed osteopenia. The serum copper level was 10.25 microgram/dl ( normal value at this age is 46 to 80 microgram/dl),serum ceruloplasmin level was 7.3 mg/dl (normal value is approximately 20 to 40 mg/dl). The MRI study of the brain revealed prominent bilateral extra-axial C.S.F. spaces with gliosis in both posterior parieto-occipital area with prominent left lateral ventricle and cerebellar folias (Figure 2). MR angiography revealed tortuosity of the cerebral vessels(Figure 3). EEG showed gross generalized polyspike waves. Opthalmoscopic examination showed pale optic disc. Microscopic examination of hair revealed classical sign of pili torti (Figure 4).

Because of lack of facility, genetic study is not possible in this case. Because copper histidinate or copper acetate is not available no definite treatment could be done. The child was treated symptomatically with anticonvulsive drugs and antibiotics for pneumonia and discharged. The parents were advised for monthly follow up.

## Discussion

Menkes disease is an X – linked disorder resulting in profound systemic copper deficiency. The Menkes disease gene is (ATP7A) encodes an enzyme p type ATPase which is required for systemic absorbtion, distribution and metabolism of copper in tissues. There is no race prediliction. The disease usually manifests at the age of 2–3 month and usually die at 3–4 years of age due to pneumonia. Our patient presented at the age of 4 months.

Molecular studies have shown gene defect and more than 150 of different mutations of the Menkes gene are reported [5,6]. The maldistribution of copper occur in different tissues e.g. accumulation in muscles, kidneys, spleen and pancreas but low in liver and brain. The serum copper concentration is low. Copper dependent enzymes like cytochrome oxidase concerned with electron transport and ATP formation, tyrosinase concerned with the pigment melanin synthesis, lysyl oxidase concerned with collagen synthesis, superoxide dismutase an antioxidant, enzyme for catecholamine biosynthesis and peptidylglycin alpha amidating monoxidase concerned with activation of neuropeptide are not functioning properly [7].

Menkes disease results in developmental delay, seizures, hypotonia which is more in trunkal muscle and feeding difficulties. All these are marked in our patient. The characteristics facies, fair complexion, chubby cheeks, sparse twisted fuzzy depigmented hair are all present in our case. The investigation finding of low serum copper, ceruloplasmin, hair structures are characteristics of Menkes disease[8]. MRI studies are supportive of the disease. Due to lack of facility genetic study is not feasible. The growth failure in our case is also a common finding of this syndrome. The low serum copper level or serum ceruloplasmin level may occur in protein energy malnutrition but the presence of pili torti, characteristics hair phenotype, asymptomatic huge bilateral subdural hematoma, and characteristics cerebral vasculature are not found in malnutrition.

We have treated the infant for bronchopneumonia and because of nonavailability and lack of sufficient data we didn't started copper histidine or copper acetate at this late presented patient of 8 months[5].

## Prevention

Genetic counseling and prenatal diagnosis are helpful though approximately 33% of cases may be due to mutaion. Recurrence risk of Menkes disease is 25% for next issue. Abnormal egress of radioactive copper in cultured amniotic cells or the cultured chorionic cells is the basis for the prenatal testing.

## Conclusion

This patient may be diagnosed as protein energy malnutrition because of less growth and developmental delay. In fact the baby has had history of hospital admission twice and diagnosed as PEM case. Clinical suspicion of possibilities may lead to correct diagnosis. The typical clinical picture of this patient is noteworthy.

## Abbreviations

PEM: Protein energy malnutrition; ATP: Adenosine triphosphate; EEG: Electroencephalography; MRI: Magnetic Resonance Imaging; MR: angiography-Magnetic Resonance angiography.

## Competing interests

The authors declare that they have no competing interests.

## Authors' contributions

AKD was responsible for patient care, follow up and drafting of paper, TNG collected data and assisted AKD to prepare manuscript, KN coordinated the study and MKG involved in he radiological investigation of the study. All authors read and approved the final manuscript.

## Consent

Written informed consent was obtained from the patient for publication of this case report and accompanying images. A copy of the written consent is available for review by the Editor – in – Chief of this Journal.

## References

[B1] Kirodian BG, Gogtay NJ, Udani VP, Kashirsagar NA (2002). Treatment of Menkes disease with parental copper histidine. Indian Pediatrics.

[B2] Danks DM, Campbell PE (1972). Menkes's kinkey hair syndrome An Inherited defect of copper absorption with widespread effects. Pediatrics.

[B3] Kodama H, Murata Y, Kabayashi M (1999). Clinical manifestations and treatment of Menkes disease and its variants. Pediatr Int.

[B4] Drank DM, Cartwright E, Stevens BJ, Townley RR Menke's kinkey hair diaease: Further definition of the defect in copper transport. Science.

[B5] Christodoulou J, Danks DM, Sarkar B (1998). Early treatment of Menkes disease with parenteral copper-histidine: long-term follow-up of four treated patients. Am J Med Jenet.

[B6] Mollar LB, Tumer Z, Lund C (2000). Similar splice-site mutation s of ATP7A gene lead to different phenotypes: classical Menkes disease or occipital horn syndrome. Am J Human Genet.

[B7] Culotta VC, Gitlin JD (2001). Disorders of copper metabolism in Scriver CR et.el. edn. The molecular and metabolic basis of inherited disease.

[B8] Koeller D, Robert D Steiner (2003). Metal metabolism disorder. Neil McIntosh, Peter Helms, Rosalind Smyth edn. Forfer and Arneil's Textbook of Pediatrics.

